# Pharmacophore annotation using extended Hückel theory

**DOI:** 10.1186/1758-2946-6-S1-P54

**Published:** 2014-03-11

**Authors:** Paul Labute, Markus Kossner, Alain Ajamian, Martin Santavy, Anna Lin

**Affiliations:** 1 Chemical Computing Group, Montreal, H3A 2R7, Canada; 2 Chemical Computing Group, Köln, 50672, Germany

## 

Pharmacophore models play an essential role in drug discovery. Generating pharmacophore models which encode accurate molecular recognition features are highly dependent on properly defined annotations. Simplistic or ill-defined pharmacophore annotations which do not capture subtle electronic or geometric effects lead to many inaccuracies. SMARTS patterns which are often used to specify annotation "rules" are subject to such inaccuracies.

The application of Extended Huckel Theory (EHT) to pharmacophore annotations compensates for deficiencies observed in "rule" based methods by taking into account electron withdrawal and resonance effects and treating these effects in a consistent manner independent of structural depiction. The application of the EHT approach will be further described and discussed through a number of case studies.

